# Humble leadership and its outcomes: A meta-analysis

**DOI:** 10.3389/fpsyg.2022.980322

**Published:** 2022-12-21

**Authors:** Yifei Luo, Zeyu Zhang, Qishu Chen, Kairui Zhang, Yijiang Wang, Jianfeng Peng

**Affiliations:** School of Labor and Human Resources, Renmin University of China, Beijing, China

**Keywords:** humble leadership, meta-analysis, outcomes, transformational leadership, servant leadership

## Abstract

The importance of humble leadership has garnered attention from both researchers and practitioners. Unfortunately, despite the accumulation of recent findings on the effects of leader humility, a quantitative review remains scant. In addressing this void, this study is among the first to conduct a meta-analytic review of humble leadership and its outcomes. Eighty-four correlations (*N* = 16,534) from 53 independent studies are synthesized. The authors found that: (a) humble leadership is positively related to affective commitment (ρ = 0.56), affective trust (ρ = 0.62), creativity (ρ = 0.39), engagement (ρ = 0.40), leader–member exchange (LMX) (ρ = 0.58), job satisfaction (ρ = 0.51), organizational identification (ρ = 0.48), psychological empowerment (ρ = 0.33), self-efficacy (ρ = 0.24), task performance (ρ = 0.33), and voice (ρ = 0.34); and that (b) humble leadership contributes a significant incremental variance beyond transformational, servant, and ethical leadership in several crucial criterion variables, providing solid evidence for the construct's uniqueness. However, humble leadership does not explain incremental variance in some criterion variables, indicating that future studies should control for the influence of some positive leadership (e.g., transformational and servant leadership). Age, gender, study design, country, and year partially moderate the correlations of interest. We discuss our findings with caution and propose future research directions.

## Introduction

Narcissistic individuals show an inflated sense of self-importance, unjustifiably high self-esteem, and low levels of empathy (Campbell et al., 2010). In the organizational field, narcissistic leaders may have been at the forefront of acquisition mistakes, cover-ups, and accounting scandals (Kelemen et al., 2022). Moreover, leader narcissism may have deleterious effects on both organizations (Resick et al., 2009; O'Reilly et al., 2018) and employees (Braun et al., 2018; Carnevale et al., 2018). As such, researchers and practitioners became intrigued by leaders' humility and humble behavior (Owens and Hekman, 2012; Owens et al., 2013; Kelemen et al., 2022) as countervailing forces toward narcissism and over-confidence.

Humble leadership, defined as “the leadership that involves viewing oneself accurately, providing an appreciation of others' strengths and contributions, and modeling teachability” (Owens et al., 2013, p. 1518), is associated with many positive employee outcomes, such as work engagement (Ma et al., 2019; Li X. et al., 2021), organizational citizenship behavior (OCB) (Ding et al., 2020; Nguyen et al., 2020), voice behavior (Li et al., 2018; Zhou et al., 2021).

Albeit the growing body of empirical evidence, some caveats impede the advancement of research and theory in humble leadership literature. First, our knowledge of the true relationship between humble leadership and its outcomes is still limited. For example, some studies found small effect sizes between humble leadership and OCB (Qin et al., 2019; Nguyen et al., 2020), while other studies found moderate ones (Cho et al., 2020; Ding et al., 2020). Statistical artifacts (such as sampling error and measure error, Hunter and Schmidt, 2004) prevent primary studies from obtaining accurate correlations of interest. For instance, when two samples come from different organizations, the correlations of interest may differ due to sampling error. A qualitative review of humble leadership (e.g., Kelemen et al., 2022) could not address this. Fortunately, the meta-analysis methodology could assist researchers in correcting erroneous statistical artifacts and evaluating the true score correlations of interest. In doing so, we seek to provide a more accurate understanding of the relationships between humble leadership and its outcomes, contributing to humble leadership literature.

Second, it is unclear whether humble leadership demonstrates incremental validity over other positive leadership styles (e.g., servant, transformational, and ethical leadership). This concern arises from the prior finding of a strong positive correlation (ρ = 0.81) between servant leadership and humble leadership (Lee et al., 2020a). Due to the high correlation between humble and transformational leadership, a large amount of variance explained by humble leadership may be explained by servant leadership. Moreover, there may be conceptual similarities between humble leadership and servant leadership. For instance, both servant and humble leaders exhibit humility and value the contributions of their followers (Dennis and Bocarnea, 2005; Liden et al., 2008; Owens et al., 2013). Beyond servant leadership, this study also examines transformational and ethical leadership. In doing so, we address the calls for empirically providing evidence of the effects of humble leadership above other leadership constructs (Kelemen et al., 2022), providing evidence for the uniqueness of humble leadership.

Finally, scholars still lack knowledge of the potential influence of moderators on the relationship between humble leadership and its outcomes. Early meta-analyses (e.g., Bal et al., 2008; Wu et al., 2018; Li P. et al., 2021; Xue et al., 2022) identified the moderating roles of age, gender, study design (cross-temporal vs. time-lagged), country, and publication year. For instance, Xue et al. (2022) found the moderating effects of year and research design when studying servant leadership–intrinsic motivation linkage; Li P. et al. (2021) found the moderation roles of study design and country when researching the relationship between leadership and engagement. The current study seeks to explore the potential moderating roles of these factors, contributing to humble leadership literature. By doing so, this study enriches our knowledge of the boundary conditions of relationships between humble leadership and its outcomes.

Taken together, we extend the humble leadership literature in three respects by addressing the aforementioned issues. The first entails estimating the true score correlations between humble leadership and its outcomes. To the best of our knowledge, our study is the first comprehensive meta-analysis on humble leadership. Obtaining a meta-analytic account of the field's current state, we attempt to clarify the relations between humble leadership and its outcomes. The second objective is systematically analyzing humble leadership's incremental variance vs. transformational, servant, and ethical leadership across various criterion measures. Using meta-analytic evidence, we aim to establish the uniqueness of humble leadership. The final goal is to detect boundary conditions between humble leadership and its outcomes, applying the meta-regression methodology.

## Theoretical background and hypotheses

### Humble leadership and its outcomes

The first research goal is to estimate the true score correlations between humble leadership and its outcomes. We will introduce humble leadership. Then, we will briefly develop the hypotheses between humble leadership and its outcomes because these hypotheses have been developed in the early primacy studies and tested yet, and our goal focuses on the accurate links between humble leadership and its outcomes. Table 1 presents the definition of the major variables in the current study.

**Table 1:** Major variables definitions.

**Variable**	**Definition**
Servant leadership	Servant leadership refers to leadership that is “(1) other-oriented approach to leadership (2) manifested through one-on-one prioritizing of follower individual needs and interests, (3) and outward reorienting of their concern for self toward concern for others within the organization and the larger community” (Eva et al., 2019, p. 114).
Ethical leadership	Ethical leadership is defined as “the demonstration of normatively appropriate conduct through personal actions and interpersonal relationships, and the promotion of such conduct to followers through two-way communication, reinforcement, and decision-making” (Brown et al., 2005, p. 120).
LMX	LMX reflects the “exchange quality between leaders and their followers. Low LMX relationships are characterized by economic exchange based on formally agreed on, immediate, and balanced reciprocation of tangible assets, such as employment contracts focusing on pay for performance; high-LMX relationships increasingly engender feelings of mutual obligation and reciprocity” (Dulebohn et al., 2012, p. 1717).
Affective commitment	Affective commitment denotes “employees' emotional attachment to, identification with, and involvement in the organization” (Meyer et al., 2002, p. 21).
Affective trust	Affective trust refers to the “emotional bonds between individuals” grounded in expressing “genuine care and concern for the welfare” of the other party (McAllister, 1995, p. 26).
Creativity	Creativity refers to the development of practical and new solutions to workplace challenges (Amabile, 1988).
Engagement	Work engagement is defined as “a positive, fulfilling, work-related state that is characterized by vigor, dedication, and absorption” (Schaufeli et al., 2002, p. 74).
Job satisfaction	Job satisfaction refers to an optional or positive emotional state arising from the appraisal of one's job or job experiences (Judge and Locke, 1993).
OCB	OCB is defined as “individual behavior that is discretionary, not directly or explicitly recognized by the formal reward system, and that in the aggregate promotes the effective functioning of the organization” (Organ, 1988, p. 4).
Organizational identification	Organizational identification refers to the perception of oneness with or belongingness to the organization (Ashforth and Mael, 1989).
Psychological empowerment	Psychological empowerment is a set of four cognitions that reflect an individual's orientation to his or her work role: meaning, competence, self-determination, and impact (Spreitzer, 1995).
Psychological safety	Psychological safety is a cognitive state in which employees “feel able to show and employ one's self without fear of negative consequences to self-image, status, or career” (Kahn, 1990, p. 708).
Self-efficacy	Self-efficacy is “concerned with judgments of how well one can execute courses of action required to deal with prospective situations” (Bandura, 1982, p. 122).
Negative affect	Negative affect is a general dimension of subjective distress and unpleasurable engagement that subsumes a variety of aversive mood states, including anger, contempt, disgust, guilt, fear, and nervousness (Watson et al., 1988, p. 1063).
Voice	Voice is defined as “nonrequired behavior that emphasizes the expression of constructive challenge with an intent to improve rather than merely criticize” (Van Dyne and LePine, 1998, p. 109).

The concept of humility is deeply rooted in both eastern and western cultures and has a lengthy history. In eastern culture, for instance, Confucius is considered an exemplar of humility (Mason, 2021), whereas, in western culture, Aristotle considers humility to be a weak virtue (Grenberg, 2005). Unethical behavior and events in the business world motivated the study of moral behavior. Owens and Hekman (2012) developed a qualitative model of humble leadership behaviors, outcomes, and contingencies in response to this trend. Subsequently, Owens et al. (2013) developed the concept and the measure of humble leadership.

In the review process, we notice that humble leadership is positively associated with task performance (Mao et al., 2018; Al Wali et al., 2022), OCB (Cho et al., 2020; Ding et al., 2020), voice (Bharanitharan et al., 2018; Li et al., 2018), creativity (Wang et al., 2016; Ye et al., 2020), leader–member exchange (LMX) (Basford et al., 2014; Wang et al., 2021), affective commitment (Basford et al., 2014; Wang et al., 2022), affective trust (Nguyen et al., 2020; Liborius and Kiewitz, 2022), job satisfaction (Owens et al., 2013; Zhong et al., 2019), psychological safety (Qian et al., 2020; Wang and Zhou, 2021), self-efficacy (Mao et al., 2018; Ma et al., 2019), and engagement (Yang et al., 2019; Nguyen et al., 2020). However, humble leadership is negatively related to turnover intention (Li et al., 2016; Liborius and Kiewitz, 2022), voluntary turnover (Owens et al., 2013; Liborius and Kiewitz, 2022), and negative affect (Basford et al., 2014). Due to statistical artifacts (e.g., sampling and measurement error), inconsistent results have been found. For instance, Bahadur and Ali (2021) found a medium correlation (*r* = 0.15) between humble leadership and task performance, whereas Cho et al. (2020) found a large one (*r* = 0.47). The current study seeks to evaluate the accurate links between humble leadership and its outcomes using the meta-analysis methodology.

### Incremental variance

The second research goal is to test the incremental validity of humble leadership over three positive leadership styles (i.e., transformational, servant, and ethical leadership). Incremental validity answers such a question: does a measure add to the prediction of a criterion above what can be predicted by other sources of data (Hunsley and Meyer, 2003)? In the humble leadership literature, Kelemen et al. (2022) suggested that “future scholarly work needs to establish where humble leadership rests within the nexus of different leadership behaviors” (p. 17). In other words, studies should probe into the incremental variance explained by humble leadership above other related leadership. Aside from the above calls to investigate the incremental validity of humble leadership, we are also motivated by the following observations. Previous studies revealed that humble leadership was highly correlated (*r* > 0.7) with the transformational, servant, and ethical leadership (Lee et al., 2020a).

Then, we have discerned commonalities between these leadership styles (i.e., transformational, servant, and ethical leadership) and humble leadership. Both transformational and humble leaders are conducive to followers' motivation. For instance, humble leaders appreciate their followers' contributions (Owens et al., 2013), while transformational leaders inspire their followers intellectually (Bass, 1999). Servant leaders tend to prioritize their followers' interests (Liden et al., 2008) and demonstrate humility toward them (van Dierendonck and Nuijten, 2010). In particular, servant leaders would learn from others, acknowledge their mistakes (van Dierendonck and Nuijten, 2010), and know their strengths and weaknesses (Dennis and Bocarnea, 2005). Thus, servant leadership is conceptually comparable to humble leadership. Like humble leaders, ethical leaders also emphasize listening to their followers (Brown et al., 2005).

Finally, although Owens et al. (2013) developed the measure of humble leadership, they did not distinguish between humble leadership and other forms of positive leadership (e.g., transformational leadership). In addition, when studying humble leadership, few primary studies controlled for the influence of other positive leadership.

In light of (a) the high correlations between humble and transformational, servant, and ethical leadership and (b) their conceptual overlaps, it is imperative to evaluate the incremental validity of humble leadership in order to determine whether it adds to the prediction of criteria beyond those explained by the transformational, servant, and ethical leadership alone. Consequently, we endeavor to respond to the following question:

***Research question 1:** Can humble leadership reflect incremental validity vis-a-vis transformational, ethical, and servant leadership when predicting important criterion variables?*

### Moderation factors

The final goal of the current study is to explore the moderating role of age, gender, study design, country, and year.

#### Age

Employees' age may play an essential role in understanding leadership effectiveness. To start, younger and older employees vary in job attitudes (Ng and Feldman, 2010) and job performance (Sturman, 2003). Thus, under the influence of the same leadership, younger and older employees may have different levels of job attitudes and performance. Second, younger and older employees may have different perceptions of the same type of leadership (McCann and Holt, 2009), making the effectiveness of leadership different. Third, older employees may hold more positive attitudes toward their leaders than their younger colleagues (Wang et al., 2019), causing them to make different ratings of the same leader. Finally, a prior meta-analysis found that age moderates the relationship between leadership and safety behavior (Liborius and Kiewitz, 2022). As the potential moderating of age is very complex, we seek to explore their relationships utilizing meta-analysis technology.

#### Gender

Humble leadership effects may vary according to followers' gender. Leadership will influence followers in the reciprocal process between leaders and followers. Early studies suggested that gender would influence leader effectiveness (Douglas, 2012; Dirik, 2020). To start, as the fundamental prototypes of leaders are different between men and women (Kiker et al., 2019), men and women may have different perceptions of the same leader (Chow and Irene, 2005). Then, men and women may have different responses to the same leader because they have different social expectations (Eagly and Wood, 2012). Finally, men and women may vary in the actual evaluations of leader effectiveness (Douglas, 2012). Although few theories could clearly explain the potential influence of gender on humble leadership and its outcomes, an explorative study may provide some insights to reveal the influence of gender.

#### Study design

We try to explore the moderation role of study design. When collecting data, many studies use a cross-temporal research design, namely, collecting independent and dependent variables at the same time point. Unfortunately, utilizing a cross-temporal rather than time-lagged research design may trigger common method bias (Podsakoff et al., 2003), distorting the magnitudes of correlations. Recent meta-analyses provide evidence to support the moderating role of study design (Kiker et al., 2019; Lyubykh et al., 2022).

#### Country

Recent meta-analyses (Kiker et al., 2019; Li P. et al., 2021; Lyubykh et al., 2022) found that the relationship between leadership and its outcomes varies according to country. However, these findings are quite inconsistent. House et al. (2004) also suggest that leadership is culturally contingent. In other words, the country may influence the relationship of interest, although their relationships could be complex. We seek to explore the moderating role of the country.

#### Year

It is quite interesting to consider the influence of publication year. Because the year may relate to many factors, such as economic conditions and research paradigms (Xue et al., 2022). It is also possible that overall study quality increases as the year increases, which may influence the correlations of interest. We want to provide exploratory evidence about the impact of the year. Taken together, the current studies seek to detect these five potential moderators, answering the following research question:

***Research question 2:** Do age, gender, study design, country, and year moderate the relationship between humble leadership and its outcome?*

## Methods

### Literature search

Following some recently-published meta-analyses (Hoch et al., 2018; Lee et al., 2020a,b), we searched the published articles associated with humble leadership by using several databases, including Web of Science and Google Scholar. According to an early study about humble leadership (Owens et al., 2013) and a recent review about humble leadership (Kelemen et al., 2022), we applied the following keywords: “*humble leadership*,” “*humble leader*,” “*leader humility*,” and “*leader expressed humility*.” We searched the abstracts, titles, and keywords of articles to locate potential articles. Data collection included studies up to April 2022.

### Inclusion criteria

**Figure 1:**
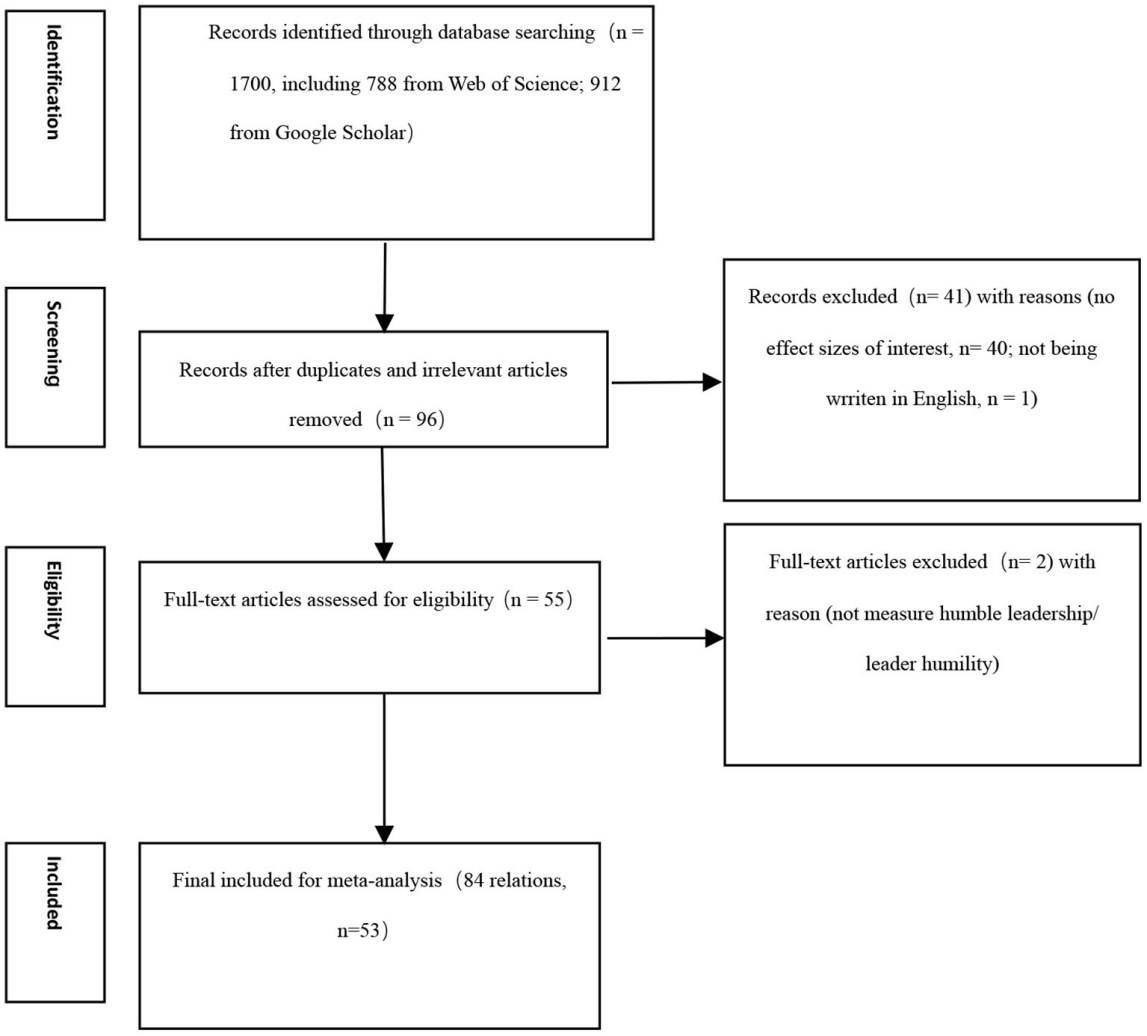
PRISMA flowchart.

To be included in our meta-analysis, the articles should meet some inclusion criteria. First, the study should be an empirical study that includes the necessary effect size(s). For instance, a qualitative study that did not include effect sizes was removed. Second, studies should be written in English. Third, data should be collected at the individual level. We noticed that some studies (e.g., Yao et al., 2021; Yao and Liu, 2022) about humble leadership are at the team level, but they do not have a sufficient number to cover a meta-analytic review. Team-level meta-analysis has more rigorous inclusion standards and needs a larger number of primary studies than individual-level meta-analysis. For instance, early team-level meta-analyses (e.g., Nicolaides et al., 2014; Lauren et al., 2016) included more than eight primary studies. The current study does not include sufficient primary studies to cover team-level meta-analysis, therefore, this meta-analysis locates at the individual level. Finally, a study should include the correlation we need. For instance, when a study does not provide the correlation we need, it would be removed. We provide a PRISMA flowchart to show our meta-analytic process (see Figure 1).

### Coding

Two authors independently coded the following information: bibliographic references (authors and publication year), sample description (sample size and country), research design/sampling strategy, effect sizes (correlations), and the reliabilities of all scales. In relation to a study that has multiple indicators of a focal construct, we averaged them (Hoch et al., 2018; Lee et al., 2020a). For instance, if one study did not report an overall correlation between a variable and humble leadership, but the correlations between dimensions of this variable and humble leadership, we averaged these correlations to evaluate an overall one. Two authors discussed the inconsistent results in the coding process until they reached an agreement.

### Publication bias analysis

Publication bias occurs because statistically significant results are published more frequently than studies without significant results (Rothstein et al., 2005). Following early meta-analyses (Baird et al., 2021; Yao et al., 2022), we used multiple ways to detect potential publication bias. In particular, we employed the Trim-and-Fill method (Duval and Tweedie, 2000) and Eggs' regression (Egger et al., 1997) to detect potential publication bias (see Table 2) using the *metafor* package (Viechtbauer, 2010) in R.

**Table 2:** Publication bias analysis.

	**Trim-and-Fill**					**Egg's regression**		
**Variable**	**Observed *k***	**Unadj. *r*+**	**Imputed *k***	**Adj. *r*+**	**Change**	***t***	***df***	***p***
Affective commitment	3	0.5	0	0.5	0	−1.96	1	0.3
Affective trust	4	0.57	1	0.54	−0.03	0.83	2	0.493
Creativity	15	0.35	0	0.35	0	−0.5	13	0.624
Engagement	7	0.39	0	0.39	0	0.56	5	0.601
Job satisfaction	2	0.44	0	0.44	0	–	–	–
LMX	5	0.51	0	0.51	0	−2.06	3	0.131
OCB	6	0.19	0	0.19	0	1.53	4	0.2
Organizational identification	5	0.43	0	0.43	0	−0.54	3	0.627
Psychological empowerment	3	0.31	0	0.31	0	6.35	1	0.099
Psychological safety	3	0.26	0	0.26	0	−0.62	1	0.649
Self-efficacy	4	0.21	0	0.21	0	−0.03	2	0.98
Task performance	7	0.33	1	0.29	−0.04	1.71	5	0.15
Turnover intention	3	−0.56	0	−0.56	0	37.08	1	0.017
Voice	13	0.28	1	0.30	0.02	−1.94	11	0.078
Voluntary turnover	2	–	–	–	–	–	–	–

Observed *k*, number of aggregated effect sizes included in analyses; Unadj. *r*+, unadjusted effect size estimate; imputed; *k*, number of additional effect sizes added by Trim-and-Fill analyses; Adj. *r*+, adjusted effect size estimate (i.e., including imputed studies).

### Meta-analytical procedures

We applied the Hunter-Schmidt method meta-analysis (Hunter and Schmidt, 2004) to calculate true score correlations of interest. This analysis was conducted in the *psychmeta* package (Dahlke and Wiernik, 2019) in R. Specifically, reliabilities (i.e., Cronbach's α) were used to correct measurement error. In line with recently-published meta-analyses (Hoch et al., 2018; Lee et al., 2020a,b), Pearson's rather than Spearman's correlation is employed as effect sizes. This is because the variance and confidence interval of Hunter and Schmidt (2004)'s meta-analysis is calculated according to Pearson's correlation. Besides, all the variables in the current study are continuous. The basic information of reliabilities is provided in Table 3. A random-effect model meta-analysis was employed to correct the sampling error. The results of the meta-analyses was presented in Table 4.

**Table 3:** Cronbach's α reliabilities of the current study.

**Variable**	**Number of α**	**Average of α**	**Maximum α**	**Minimum α**	**Sample size weighted average of α**
Humble leadership	84	0.92	1	0.73	0.92
Affective commitment	3	0.92	0.97	0.85	0.91
Affective trust	4	0.83	0.93	0.66	0.84
Creativity	15	0.9	0.97	0.85	0.87
Engagement	7	0.92	0.95	0.83	0.92
Job satisfaction	2	0.78	0.82	0.75	0.8
LMX	5	0.88	0.91	0.88	0.89
OCB	6	0.84	0.9	0.69	0.87
Organizational identification	5	0.87	0.88	0.85	0.87
Psychological empowerment	3	0.91	0.94	0.85	0.91
Psychological safety	3	0.81	0.95	0.73	0.79
Self-efficacy	4	0.85	0.91	0.82	0.84
Task performance	7	0.83	0.96	0.7	0.83
Negative affect	2	0.89	0.93	0.85	0.9
Turnover intention	3	0.81	0.88	0.78	0.8
Voice	13	0.87	0.93	0.76	0.87
Voluntary turnover	2	1	1	1	1

**Table 4:** Bivariate relationships between humble leadership and its outcomes.

**Variable**	***k***	***n***	***r***	**ρ**	**SDρ**	**95% CI**	**80% CR**
Affective commitment	3	1,439	0.52	0.56	0.18	[0.10, 1.02]	[0.21, 0.90]
Affective trust	4	999	0.55	0.62	0.19	[0.31, 0.93]	[0.31, 0.92]
Creativity	15	5,071	0.35	0.39	0.18	[0.29, 0.49]	[0.15, 0.63]
Engagement	7	2,432	0.37	0.4	0.14	[0.26, 0.54]	[0.19, 0.61]
Job satisfaction	2	932	0.44	0.51	0	[0.20, 0.81]	[0.51, 0.51]
LMX	5	1,863	0.53	0.58	0.22	[0.31, 0.86]	[0.25, 0.92]
OCB	6	1,491	0.14	0.16	0.19	[−0.05, 0.37]	[−0.12, 0.43]
Organizational identification	5	1,212	0.43	0.48	0.15	[0.28, 0.68]	[0.25, 0.71]
Psychological empowerment	3	917	0.31	0.33	0.1	[0.06, 0.61]	[0.15, 0.51]
Psychological safety	3	732	0.27	0.31	0.14	[−0.08, 0.71]	[0.04, 0.59]
Self-efficacy	4	1,367	0.21	0.24	0.11	[0.04, 0.45]	[0.06, 0.43]
Task performance	7	1,439	0.29	0.33	0.16	[0.17, 0.49]	[0.10, 0.56]
Negative affect	2	786	−0.05	−0.06	0.17	[−1.68, 1.56]	[−0.58, 0.47]
Turnover intention	3	669	−0.57	−0.65	0.29	[−1.38, 0.08]	[−1.20, −0.11]
Voice	13	4,050	0.31	0.34	0.19	[0.22, 0.46]	[0.08, 0.60]
Voluntary turnover	2	814	−0.17	−0.17	0.09	[−1.12, 0.78]	[−0.46, 0.12]

*k*, number of studies; *n*, total sample size in the meta-analysis; *r*, uncorrected effect size; ρ, corrected effect size; SDρ, standard deviation of the corrected effect size; CI, confidence interval; CV, credibility interval.

### Incremental variance analysis

To accomplish incremental analysis, based on the results of this study and recently-published meta-analysis (e.g., Hoch et al., 2018), we built a table that included a series of necessary correlation coefficients (see Table 5). Then, we applied these correlations to conduct the incremental variance analysis. In particular, we added humble leadership to the model after controlling transformational, servant, and ethical leadership, in order to see whether humble leadership could explain incremental variance after controlling transformational, servant, and ethical leadership. Consistent with the suggestion by Viswesvaran and Ones (1995), we used the harmonic mean of the different correlations to conduct these analyses. This analysis was conducted using MPLUS (Muthén and Muthén, 2017). The results are shown in Table 6.

**Table 5:** Meta-analytic correlation required for incremental analysis.

**Variable**	***k***	***n***	***r***	**ρ**	**SDρ**	**95% CI**
HL-TL^a^	3	497	0.73	0.8	0.16	–
HL-SL^a^	1	283	–	0.81	–	–
HL-EL^a^	2	545	0.75	0.79	0.12	–
TL-SL^b^	5	774	0.47	0.52	0.08	[0.45, 0.60]
TL-EL^b^	20	3,717	0.63	0.7	0.17	[0.62, 0.79]
SL-EL^d^	4	3,106	0.74	0.82	0.11	–
HL-affective commitment	3	1,439	0.52	0.56	0.18	[0.10, 1.02]
HL-affective trust	4	999	0.55	0.62	0.19	[0.31, 0.93]
HL-creativity	15	5,071	0.35	0.39	0.18	[0.29, 0.49]
HL-engagement	7	2,432	0.37	0.4	0.14	[0.26, 0.54]
HL-job satisfaction	2	932	0.44	0.51	0	[0.20, 0.81]
HL-LMX	5	1,863	0.53	0.58	0.22	[0.31, 0.86]
HL-TASK performance	7	1,439	0.29	0.33	0.16	[0.17, 0.49]
HL-voice	13	4,050	0.31	0.34	0.19	[0.22, 0.46]
TL-affective commitment^b^	30	11,835	0.36	0.42	0.16	[0.36, 0.48]
TL-affective trust^b^	23	7,048	0.56	0.65	0.17	[0.56, 0.72]
TL-creativity^a^	55	18,122	0.28	0.31	0.2	[0.23, 0.33]
TL-engagement^b^	14	5,300	0.44	0.48	0.27	[0.35, 0.63]
TL-job satisfaction^b^	55	20,344	0.37	0.42	0.2	[0.37, 0.47]
TL-LMX^b^	20	4,591	0.64	0.71	0.18	[0.63, 0.80]
TL-task performance^b^	74	18,129	0.25	0.27	0.15	[0.24, 0.31]
TL-voice^c^	13	6,204	0.27	0.3	0.06	[0.26, 0.34]
SL-affective commitment^b^	5	1,436	0.35	0.41	0.27	[0.18, 0.65]
SL-affective trust^b^	7	1,886	0.63	0.71	0.12	[0.58, 0.82]
SL-creativity^a^	11	4,490	0.34	0.38	0.25	[0.21, 0.47]
SL-engagement^b^	4	959	0.47	0.52	0	[0.47, 0.58]
SL-job satisfaction^b^	11	2,671	0.6	0.66	0.11	[0.59, 0.73]
SL-LMX^b^	4	938	0.59	0.65	0.18	[0.58, 0.82]
SL-task performance^b^	8	2,077	0.2	0.23	0.08	[0.15, 0.31]
SL-voice^d^	7	1,797	0.23	0.25	0.14	–
EL-affective commitment^b^	24	4,873	0.42	0.48	0.15	[0.41, 0.55]
EL-affective trust^b^	18	4,105	0.58	0.66	0.27	[0.54, 0.79]
EL-creativity^a^	15	3,982	0.31	0.36	0.14	[0.24, 0.39]
EL-engagement^b^	6	1,335	0.35	0.39	0.1	[0.29, 0.48]
EL-job satisfaction^b^	17	4,578	0.45	0.5	0.2	[0.44, 0.56]
EL-LMX^b^	18	4,052	0.65	0.71	0.2	[0.63, 0.81]
EL-task performance^b^	22	4,904	0.22	0.25	0.06	[0.21, 0.29]
EL-voice^c^	4	1,846	0.19	0.21	0.02	[0.18, 0.24]

HL, humble leadership; TL, transformational leadership; SL, servant leadership; EL, ethical leadership; Unless stated, meta-analytic correlations were calculated by the authors:

a Lee et al. (2020a),

b Hoch et al. (2018),

c Chamberlin et al. (2017),

d Lee et al. (2020b).

**Table 6:** Incremental variance analysis.

	***N***	***B***	***SE***	***R^2^***	***B***	***SE***	***R^2^***	**Δ*R*^2^**	**Δ*R*^2^%**
**Affective commitment**									
TL	914	0.173^**^	0.04	0.246	−0.295^**^	0.05	0.354	0.108	30.51%
SL		0.079	0.05		−0.423^**^	0.06			
EL		0.294^**^	0.06		0.356^**^	0.06			
HL					0.858^**^	0.07			
**Affective trust**									
TL	898	0.431^**^	0.03	0.616	0.815^**^	0.05	0.688	0.072	10.47%
SL		0.586^**^	0.03		0.998^**^	0.04			
EL		−0.122^**^	0.04		−0.173^**^	0.04			
HL					−0.704^**^	0.05			
**Creativity**									
TL	1,001	0.144^**^	0.04	0.162	0.053	0.06	0.166	0.004	2.41%
SL		0.283^**^	0.05		0.185^**^	0.07			
EL		0.028	0.06		0.04	0.06			
HL					0.167^*^	0.08			
**Engagement**									
TL	858	0.479^**^	0.04	0.389	0.996^**^	0.04	0.52	0.131	25.19%
SL		0.69^**^	0.04		1.244^**^	0.05			
EL		−0.511^**^	0.05		−0.579^**^	0.05			
HL					−0.947^**^	0.06			
**Job satisfaction**									
TL	911	0.222^**^	0.03	0.465	0.482^**^	0.05	0.499	0.034	6.81%
SL		0.8^**^	0.04		1.078^**^	0.06			
EL		−0.311^**^	0.05		−0.345^**^	0.05			
HL					−0.476^**^	0.06			
**LMX**									
TL	883	0.447	0.03	0.618	0.959^**^	0.03	0.747	0.129	17.27%
SL		0.281	0.04		0.829^**^	0.04			
EL		0.167	0.04		0.099^**^	0.04			
HL					−0.937^**^	0.04			
**Task performance**									
TL	935	0.198^**^	0.04	0.084	−0.049	0.6	0.114	0.03	26.32%
SL		0.109^*^	0.06		−0.155^*^	0.07			
EL		0.02	0.07		0.055	0.07			
HL					0.451^**^	0.08			
**Voice**									
TL	929	0.331^**^	0.04	0.117	0.162^**^	0.06	0.131	0.014	10.69%
SL		0.292^**^	0.05		0.111	0.07			
EL		−0.261^**^	0.07		−0.239^**^	0.07			
HL					0.308^**^	0.08			

HL, humble leadership; TL, transformational leadership; SL, servant leadership; EL, ethical leadership; *N*, harmonic mean.

** *p* < 0.01.

* *p* < 0.05.

### Moderation analysis

Random effect meta-regression technology was applied to detect potential moderators. First, the coding information was recoded into useable variables. In particular, age, gender (%female), and year were regarded as continuous variables. The country was coded as a dummy variable. Eastern country was coded as “1” whereas western was coded as “0.” For the study design, the cross-temporal research design was coded as “0” whereas the time-lagged research design was coded as “1.” Second, we employed regarded Restricted Maximum Likelihood (REML) method as an estimator to conduct our meta-regression. Such analysis was conducted in the *metafor* (Viechtbauer, 2010) package in R. The results are presented in Table 7.

**Table 7:** Moderation analysis.

**Variable**	**Moderator**	**Estimate**	***t***	***p***	**Moderator effect present?**
Affective commitment	Age	−0.075	−1.752	0.330	No
	Gender	0.009	1.746	0.331	No
	Study design	−0.383	−2.525	0.240	No
	Country	0.061	0.148	0.907	No
	Year	0.002	0.032	0.980	No
Affective trust	Age	0.039	1.127	0.377	No
	Gender	0.018	1.696	0.232	No
	Study design	−0.129	−0.422	0.714	No
	Country	−0.241	−1.111	0.382	No
	Year	0.084	0.866	0.478	No
Creativity	Age	−0.013	−1.985	0.069	Yes, the larger the age, the smaller the correlation
	Gender	0.002	0.712	0.489	No
	Study design	−0.303	−2.551	0.024	Yes, the correlation is larger when using a cross-temporal rather than time-lagged research design
	Country	–	–	–	–
	Year	0.041	1.326	0.208	No
Engagement	Age	−0.005	−0.264	0.802	No
	Gender	−0.003	−0.822	0.448	No
	Study design	0.241	2.966	0.031	Yes, the correlation is smaller when using a cross-temporal rather than time-lagged research design
	Country	0.020	0.135	0.898	No
	Year	0.024	1.026	0.352	No
LMX	Age	0.121	1.533	0.200	No
	Gender	0.007	0.552	0.610	No
	Study design	0.274	1.076	0.342	No
	Country	−0.579	−2.679	0.055	Yes, the correlation is smaller when data is collected from eastern rather than non-eastern countries
	Year	−0.062	−1.270	0.273	No
Organizational identification	Age	−0.031	−0.750	0.508	No
	Gender	−0.018	−2.993	0.058	Yes, the correlation is smaller when the proportion of females increases
	Study design	0.258	1.234	0.305	No
	Country	–	–	–	–
	Year	−0.040	−0.891	0.439	
Task performance	Age	0.035	3.201	0.024	Yes, the larger the age, the larger the correlation
	Gender	−0.005	−1.057	0.339	No
	Study design	−0.241	−1.855	0.123	No
	Country	–	–	–	–
	Year	0.136	2.794	0.038	Yes, the larger the year, the larger the correlation
Voice	Age	−0.027	−1.342	0.207	No
	Gender	0.004	1.210	0.252	No
	Study design	−0.156	−1.337	0.208	No
	Country	–	–	–	–
	Year	−0.040	−1.010	0.334	No

## Results

### Publication bias analysis

As depicted in Table 2, the overall publication bias is not serious. First, Egg's regression did not find any publication bias. That is, all the *p*-values are bigger than 0.05. Second, the Trim-and-Fill method helps to fill asymmetric effect sizes and provides an adjusted overall effect size. For the majority of variables, except for affective trust and task performance, the Trim-and-Fill method did not find an asymmetric effect size. That is, *Imputed k* equals zero. In relation to affective trust, after imputing one miss effect size, r only decreased by 0.03. For task performance, after imputing one missed correlation, r only decreased by 0.04. Together, the overall publication bias is not serious, drawing on Egg's regression and Trim-and-Fill methods, suggesting our meta-analysis could move forward.

### True score correlations

As shown in Table 4, significant (i.e., 95% CI excludes zero) and positive links have been found between humble leadership and affective commitment (ρ = 0.56), affective trust (ρ = 0.62), creativity (ρ = 0.35), engagement (ρ = 0.40), job satisfaction (ρ = 0.51), LMX (ρ = 0.58), organizational identification (ρ = 0.48), psychological empowerment (ρ = 0.33), self-efficacy (ρ = 0.24), task performance (ρ = 0.33), and voice (ρ = 0.34). However, insignificant relationships (i.e., 95% CI includes zero) have been found between humble leadership and OCB, psychological safety, negative affect, turnover intention, and voluntary turnover.

### Incremental variance analysis

As illustrated in Table 6, mixed results were found about the incremental variance explained by humble leadership. On the one hand, humble leadership contributes considerable incremental variance after controlling transformational, servant, and ethical leadership in some criterion variables (i.e., affective commitment, creativity, task performance, and voice). For instance, in relation to affective commitment, when adding humble leadership to the regression model, the model explains more than 30.51% variance above transformational, servant, and ethical leadership.

On the other hand, however, for some criterion variables (i.e., affective trust, engagement, job satisfaction, and LMX), humble leadership did not explain meaningful incremental variance after controlling the transformational, servant, and ethical leadership. For instance, when it comes to affective commitment, when humble leadership was added to the model, path coefficient *(B)* was −0.704, which is smaller than zero, suggesting that humble leadership did not contribute meaningful variance after considering the influence of the transformational, servant, and ethical leadership.

### Moderation analysis

The moderating effect is shown in Table 7. For age, the findings are mixed. The correlation between humble leadership and creativity is smaller when age is larger. However, the correlation between humble leadership and task performance is larger when age is larger. Gender only moderates the link between humble leadership and organizational identification. In particular, their correlations decrease as gender (%female) increases. The study design has a mixed effect. For creativity, the correlation is larger when utilizing a cross-temporal rather than time-lagged research design. However, for engagement, the effect of study design is the opposite. The country only moderates the humble leadership–LMX linkage, such that the correlation is smaller when data is collected from eastern rather than western countries. As the year increases, the correlation between humble leadership and task performance increases.

## Discussion

This study provides the first meta-analytic review of humble leadership literature. The authors seek to contribute to humble leadership literature by (a) evaluating the true score correlations between humble leadership and its outcome, (b) estimating the incremental variance explained by humble leadership after controlling transformational, servant, and ethical leadership, and (c) detecting the potential moderating role of age, gender, study design, country, and year. The Discussion part will focus on these three research goals. Besides, we will present the management implications, limitations, and future research directions.

### Ture score correlations of interest

To start, we find humble leadership is positively related to affective commitment (ρ = 0.56), affective trust (ρ = 0.62), creativity (ρ = 0.35), engagement (ρ = 0.40), job satisfaction (ρ = 0.51), LMX (ρ = 0.58), organizational identification (ρ = 0.48), psychological empowerment (ρ = 0.33), self-efficacy (ρ = 0.24), task performance (ρ = 0.33), and voice (ρ = 0.34), suggesting that humble leadership has good criterion-related validity in relation to a range of key employee outcomes. Our study provides accurate estimations of true score correlations between humble leadership and its outcomes after correcting statistical artifacts (i.e., measurement and sampling error), helping scholars to understand the extent to which humble leadership is related to its outcomes. Cohen (2013) provides a standard to understand the magnitude of effect sizes. That is, the small effect size is 0.1, the moderate is 0.3, and the large is 0.5. Applying this standard, we find that humble leadership has moderate to large correlations with its key outcomes.

Interestingly, humble leadership has similar correlations with creativity (ρ = 0.35) and voice (ρ = 0.34), while servant leadership also has similar correlations with task performance (ρ = 0.25) and voice (ρ = 0.25) (Lee et al., 2020b), showing that similar theoretical mechanisms may exist between humble (servant) leadership and voice and creativity. In relation to engagement, humble (ρ = 0.40), transformational (ρ = 0.43), and authentic leadership (ρ = 0.42) (Li P. et al., 2021) exhibit similar magnitude of correlations with engagement. In regard to LMX, humble leadership shows a lower correlation with LMX (ρ = 0.58) than the transformational (ρ = 0.71), servant (ρ = 0.65), ethical (ρ = 0.71), and authentic (ρ = 0.67) leadership (Hoch et al., 2018). That is to say, humble leaders may have a lower quality of social exchange relationships with their followers than the transformational, servant, ethical, and authentic leaders.

Then, insignificant relationships have been found between humble leadership and OCB, psychological safety, negative affect, turnover intention, and voluntary turnover. In relation to OCB, although we hypothesized humble leadership is positively related to OCB, their relations could be complex in some situations. For instance, Qin et al. (2019) found that negative affect mediates the links between supervisor-employee congruence in humility and employee OCB. Regarding psychological safety, consistent with previous studies (Wang et al., 2018; Qian et al., 2020), we theoretically believe that humble leadership may be positively correlated with it. However, due to the small sample size (*k* = 2), the confidence interval could be too wide to include zero. For turnover intention and voluntary turnover, although we hypothesized that they would be negatively related to humble leadership, the influence of humble leadership on turnover intention and voluntary turnover may be dependent on boundary conditions. For example, employees may want to leave their employees when they are in a highly competitive environment that requires transformational rather than humble leadership. For negative affect, one possible explanation is that humble leadership may trigger negative affect in some situations. Together, insignificant findings do not make us frustrated. On the contrary, it brings us some meaningful directions for future research. That is, future research can draw some insight from our inconsistent results, find some research gaps, and then conduct primary studies to verify them.

Finally, as OCB and task performance are measured by leaders or employees, we conducted sub-analyses to detect the potential influence of raters. The results show that the influence of raters on OCB (*F* = 0.106, *p* > 0.05) and task performance (*F* = 0.689, *p* > 0.05) are not significant. As the majority of primary studies (98%) utilize Owens et al. (2013)'s measurement to rate humble leadership, it seems the measurement of humble leadership may not influence the robustness of results. Besides, we should point out that all humble leadership included in the current study is rated by the followers. Such results may be influenced by the personality of followers (Wang et al., 2019) and are different from self-reported leadership behavior.

### Incremental variance

First, although we showed the similarities between humble leadership and transformational, servant, and ethical leadership in the Hypotheses part, we found that humble leadership contributes considerable incremental variance after controlling transformational, servant, and ethical leadership in some important criterion variables. Scholars tried to demonstrate incremental predictive validity of expressed humility over some personality traits (e.g., generalized self-efficacy and conscientiousness; Owens et al., 2013). Our study extends the boundaries of previous research on the leadership field and eliminates concerns about the conceptual redundancy of humble leadership to some extent. Our studies also respond to the research suggestions by Kelemen et al. (2022), as they recommended establishing where humble leadership rests within the nexus of different leadership behaviors.

Second, it is worth mentioning that in some criterion variables (i.e., affective trust, engagement, job satisfaction, and LMX), humble leadership does not explain meaningful variance after controlling the transformational, servant, and ethical leadership. These findings may challenge some of the existing results. Taking engagement as an example, we notice that when studying the effect of humble leadership, many studies (e.g., Yang et al., 2019; Nguyen et al., 2020) did not control any positive leadership (e.g., transformational leadership). We have reasons to challenge these findings because when controlling some forms of positive leadership (e.g., transformational leadership), significant results produced by humble leadership may become insignificant. If so, perhaps engagement is not caused by humble leadership but by other forms of positive leadership (e.g., transformational leadership). Overall, future studies need to control other positive leadership when researching the unique effect of humble leadership on affective trust, engagement, job satisfaction, and LMX.

Third, in the current meta-analytic review, humble leadership is rated by their followers. However, employees' rating of leadership is influenced by three sections, namely, actual leadership, employees' perception, and random measurement error (Lance, 1994). Wang et al. (2019) found the followers' characteristics (e.g., gender, personality) will influence the perception of leadership and thereby influence rating results. For instance, agreeable employees who are friendly and tolerant (Barrick and Mount, 1991) are likely to rate a high score for a positive leader. Therefore, if agreeable employees rate both servant and humble leadership, the actual variance explained by humble and servant leadership may be disturbed by employees' characteristics. To address this, Wang et al. (2019) suggested that employees' characteristics (e.g., personality) should be controlled. In other words, if future primary studies seek to evaluate the incremental variance of humble leadership, they should control personality.

Finally, when making the incremental variance analysis, very high correlations between humble leadership and transformational leadership (ρ = 0.80), servant leadership (ρ = 0.81), and ethical leadership (ρ = 0.79) are utilized. As we introduced in the Hypothesis part, conceptual overlaps between these leadership styles might be a reason. However, an alternative explanation still exists. Valence-based conflation, which reflects situations in which otherwise quite distinct behaviors are clustered and labeled as being part of a style simply because of shared valence, may also lead to this phenomenon (Fischer and Sitkin, 2022). When judging positive leaders (e.g., transformational and humble leaders), due to the valence-based conflation, raters (who usually are followers), may valence and conflate leader behaviors with underlying intentions, quality of execution, and realized effects. In fact, they may appraise leaders according to what good and bad leaders do rather than what leaders actually do (Fischer and Sitkin, 2022). Drawing the current measurement of these leadership styles, such errors may not be easily addressed. However, if this explanation could not be ruled out, the results of incremental variance analysis based on the current measurement should be understood with caution.

### Moderating analysis

#### Age

Age has mixed impacts of age on humble leadership–creativity and humble leadership–task performance linkage. However, these findings should be understood with caution. Actually, the impact of age on work could be complex. For example, Sturman (2003) found age has a curve relationship with job performance. Although our findings provide preliminary evidence for understanding age's impact on humble leadership and its outcome, as Ng and Feldman (2010) suggested, an advanced theory is required to understand the influence of age on work.

#### Gender

We found the correlation between humble leadership and organizational identification decrease as gender (% female) increases. Utilizing meta-analysis methodology, Riketta (2005) found a negative relationship between the female gender and organizational commitment. One possible explanation is that female is more modest than male (Fang et al., 2019) so they may report a lower level of organizational commitment.

#### Study design

For creativity, the correlation is larger when utilizing a cross-temporal rather than time-lagged study design. However, for engagement, the effect of study design is the opposite. These findings are in line with a recently-published meta-analysis (Li P. et al., 2021), as they also observed the opposite role of the study design. Although utilizing a cross-temporal design will trigger common method bias (Podsakoff et al., 2003), it is still unclear whether it will increase the correlation of interest. We also consider the potential influence of raters. In the research design process, scholars should choose to use self-reported or other-reported designs. For humble leadership, in the major primary studies, followers rated leaders' humble leadership. However, for performance (i.e., task performance and OCB), some studies used employee-reported designs whereas some used leader-reported designs. We made an explanatory moderation analysis to check the potential influence of raters. However, we did not find any significant results. We presented this analysis in the Supplementary material to provide evidence for future studies.

#### Country

Country moderates the humble leadership–LMX linkage, such that the correlation is smaller when data is collected from eastern rather than western countries. As Kelemen et al. (2022) suggest, humble leadership which shows modesty to the followers may have a weaker influence in eastern countries. To further detect the potential influence of culture, we made a supplemental analysis of one cultural indicator (i.e., individualism). We do not find any significant results. These results are presented in the Supplementary material. Given that cross-cultural studies of humble leadership are still rare, which is wealthy to be considered in the future.

#### Year

We found that as the year increases, the correlation between humble leadership and task performance increases. Interestingly, Xue et al. (2022) also found the correlation between servant leadership (ethical leadership) and intrinsic motivation increases, as the year increases. However, these findings should be understood carefully, as publication year may relate to a series of factors (e.g., management level and economic condition).

### Management implications

Our study has also generated several important implications for management practice. First, our study helps managers and employees to increase their knowledge about humble leadership. Our findings confirmed the positive relationships between humble leadership and a series of employees' attitudes (e.g., affective commitment and job satisfaction). That is, under the influence of humble leadership, employees may exhibit positive attitudes toward the organization. Second, we find humble leadership has moderate to large correlations with a series of important behavior and performance outcomes (e.g., task performance, creativity, and voice). In relation to organizations, it is crucial to develop leaders' humility. For instance, organizations could use human resource practices to improve their leaders' humility. In the training process, the human resource department could teach leaders to understand that leader humility is critical for improving their effectiveness. Besides, the human resource department could employ internal or external trainers to increase leaders' humility.

## Limitations and recommendations for future research

Some limitations should be mentioned in our meta-analysis. The first one is that our study only collects studies wring in English. Although publication bias does find serious publication bias, cultural differences may exist. Future studies could try to collect data from other languages to estimate the influence of culture. Second, this study does not evaluate the mediating effect between humble leadership and its outcomes. When more evidence is accumulated, future studies could systematically estimate the mediating effects between humble leadership and its outcomes.

Finally, this study does not include unpublished data. Although publication bias analysis showed that the potential influence of publication bias is not serious, when more evidence is accumulated, an updated meta-analysis about humble leadership could try to locate unpublished studies, providing a more comprehensive understanding of humble leadership.

## Conclusions

Humble leadership has drawn so much academic attention recently. However, a qualitative review is still lacking in this field, limiting the continuous development of humble leadership literature. This study uses the Hunter-Schmidt method meta-analysis technology to estimate the links between humble leadership and its outcomes. Humble leadership has been found to be significantly related to a series of important employee outcomes (e.g., creativity, task performance, and voice). When controlling transformational, servant, and ethical leadership, humble leadership explains considerable incremental variance in many criterion variables, but not all, suggesting future studies about humble leadership should control relevant leadership styles. Age, gender, study design, country, and year partially moderate the humble leadership–its outcomes associations. We provide management implications of humble leadership, drawing on our findings. Hoping our efforts will attract scholarly attention to the ongoing research on humble leadership.

## Data availability statement

The original contributions presented in the study are included in the article/Supplementary material, further inquiries can be directed to the corresponding author/s.

## Author contributions

YL: idea, introduction, hypotheses, method, results, and discussion. ZZ and QC: hypotheses and discussion. KZ and YW: method and discussion. JP: idea. All authors contributed to the article and approved the submitted version.
