# Supplementary material for: Humble leadership and its outcomes: A meta-analysis
Source: Front Psychol. 2022 Dec 21;13:980322. doi: 10.3389/fpsyg.2022.980322 (PMC9811147; doi:10.3389/fpsyg.2022.980322)
Supplement: Supplementary file 1 [file Data_Sheet_1.docx]

**Table S1 Supplemental Moderation Analysis of Individualism**

| variable | moderator | estimate | t-val | p-val | Moderator effect present? |
| --- | --- | --- | --- | --- | --- |
| Affective commitment | individualism | 0.005 | 2.525 | 0.240 | No |
| Affective trust | individualism | -0.005 | -1.590 | 0.253 | No |
| Creativity | individualism | -0.003 | -0.193 | 0.850 | No |
| Engagement | individualism | 0.000 | -0.145 | 0.891 | No |
| LMX | individualism | 0.008 | 2.315 | 0.104 | No |
| Organizational identification | individualism | 0.026 | 1.235 | 0.305 | No |
| Task performance | individualism | 0.034 | 1.018 | 0.355 | No |
| voice | individualism | -0.004 | -0.518 | 0.615 | No |

Notes. The individualism index was extracted from Hofstede’s website ([www.geerthofstede.com](http://www.geerthofstede.com)).

**Table S2 Supplemental Moderation Analysis of Rater**

|  | moderator | estimate | t-val | p-val | Moderator effect present? |
| --- | --- | --- | --- | --- | --- |
| OCB | rater | -0.109 | -0.649 | 0.552 | No |
| Task Performance | rater | -0.201 | -1.536 | 0.185 | No |

Notes. Self-reported is coded as “0”, whereas leader-reported is coded as “1”.

**Table S3 Major Coding Information**

| **name** | **year** | **n** | **r** | **α1** | **construct2** | **α2** | **cross-temporal** | **country** |
| --- | --- | --- | --- | --- | --- | --- | --- | --- |
| Patrick Liborius | 2022 | 196 | 0.65 | 0.94 | Affective trust | 0.66 | 1 | German |
| Patrick Liborius | 2022 | 168 | -0.24 | 0.94 | Turnover intention | 0.88 | 1 | German |
| Patrick Liborius | 2022 | 110 | -0.35 | 0.94 | Voluntary turnover | 1 | 1 | German |
| Xingyu Wang | 2022 | 330 | 0.32 | 0.96 | Affective commitment | 0.97 | 1 | China |
| Joather Al Wali | 2021 | 173 | 0.462 | 0.984 | Creativity | 0.944 | 0 | Malaysia |
| Joather Al Wali | 2021 | 173 | 0.369 | 0.984 | Creativity | 0.944 | 0 | Malaysia |
| Joather Al Wali | 2021 | 173 | 0.488 | 0.984 | Task performance | 0.964 | 0 | Malaysia |
| Shanshan Qian | 2020 | 241 | 0.38 | 0.92 | Psychological safety | 0.73 | 0 | China |
| Rahmi Bhatia | 2020 | 252 | 0.58 | 0.93 | Organizational identification | 0.87 | 0 | Egypt |
| Rahmi Bhatia | 2020 | 252 | -0.68 | 0.93 | Turnover intention | 0.78 | 0 | Egypt |
| Yuan-Yuan LIU | 2016 | 213 | 0.65 | 0.89 | Voice | 0.83 | 0 | China |
| Chao Ma | 2020 | 449 | 0.57 | 0.91 | Engagement | 0.95 | 1 | China |
| Chao Ma | 2020 | 185 | 0.3 | 0.91 | Engagement | 0.93 | 1 | China |
| Chao Ma | 2020 | 449 | 0.12 | 0.91 | Self -efficacy | 0.83 | 1 | China |
| Chao Ma | 2020 | 449 | 0.15 | 0.91 | voice | 0.91 | 1 | China |
| Chao Ma | 2020 | 185 | 0.1 | 0.91 | Voice | 0.76 | 1 | China |
| Peng Wang | 2021 | 496 | 0.49 | 0.93 | LMX | 0.88 | 0 | China |
| Juan Wang | 2016 | 451 | 0.17 | 0.9 | Creativity | 0.972 | 0 | China |
| Jeewon Cho | 2020 | 233 | 0.375 | 0.93 | OCB | 0.81 | 0 | China |
| Jeewon Cho | 2020 | 233 | 0.47 | 0.93 | Task performance | 0.8 | 0 | China |
| Waseem Bahadur | 2020 | 294 | 0.15 | 0.9 | Task performance | 0.81 | 1 | China |
| Tessa E. Basford | 2014 | 511 | 0.47 | 0.93 | Affective Commitment | 0.85 | 1 | US |
| Tessa E. Basford | 2014 | 511 | 0.76 | 0.93 | LMX | 0.92 | 1 | US |
| Tessa E. Basford | 2014 | 511 | 0.03 | 0.93 | Negative affect | 0.93 | 1 | US |
| K. Bharanitharan | 2019 | 257 | 0.24 | 0.87 | Self -efficacy | 0.7 | 0 | India |
| K. Bharanitharan | 2019 | 257 | 0.245 | 0.87 | Voice | 0.77 | 0 | India |
| Long Chen | 2021 | 237 | 0.16 | 0.93 | Voice | 0.84 | 0 | China |
| Jie Li | 2016 | 249 | 0.58 | 0.93 | Organizational identification | 0.87 | 1 | China |
| Jie Li | 2016 | 249 | -0.68 | 0.93 | Turnover intention | 0.78 | 1 | China |
| Xiaokai Li | 2021 | 237 | 0.28 | 0.83 | Creativity | 0.92 | 0 | China |
| Xiaokai Li | 2021 | 237 | 0.3 | 0.83 | Engagement | 0.83 | 0 | China |
| Huiyue Diao | 2019 | 200 | 0.18 | 0.93 | Task performance | 0.93 | 1 | China |
| Yanfei Wang | 2018 | 328 | 0.27 | 0.88 | Psychological safety | 0.76 | 1 | China |
| Yanhan Zhu | 2019 | 278 | 0.3 | 0.91 | Affective trust | 0.81 | 1 | China |
| He Ding | 2020 | 260 | 0.3 | 0.96 | OCB | 0.92 | 1 | China |
| Diep T. N. Nguyen | 2020 | 252 | 0.64 | 0.96 | Affective trust | 0.92 | 1 | Australia |
| Diep T. N. Nguyen | 2020 | 252 | 0.5 | 0.96 | Engagement | 0.93 | 1 | Australia |
| Diep T. N. Nguyen | 2020 | 252 | 0.05 | 0.96 | OCB | 0.9 | 1 | Australia |
| Zheng Zhang | 2021 | 163 | 0.12 | 0.97 | Psychological safety | 0.95 | 1 | China |
| Zheng Zhang | 2021 | 163 | 0.07 | 0.97 | Voice | 0.94 | 1 | China |
| Farwa Asghar | 2022 | 405 | 0.433 | 0.9 | Creativity | 0.89 | 0 | Pakistan |
| Farwa Asghar | 2022 | 405 | 0.335 | 0.9 | Self -efficacy | 0.82 | 0 | Pakistan |
| Wasim Abbas | 2019 | 498 | 0.485 | 0.858 | Creativity | 0.846 | 0 | Pakistan |
| Jianghua Mao | 2019 | 256 | 0.15 | 0.92 | Self-efficacy | 0.91 | 0 | China |
| Jianghua Mao | 2019 | 256 | 0.18 | 0.92 | Task performance | 0.85 | 0 | China |
| Kejian Yang | 2019 | 377 | 0.34 | 0.92 | Creativity | 0.92 | 0 | China |
| Kejian Yang | 2019 | 377 | 0.27 | 0.92 | Engagement | 0.92 | 0 | China |
| Joel B. Carnevale | 2019 | 233 | 0.18 | 0.93 | LMX | 0.91 | 0 | China |
| Joel B. Carnevale | 2019 | 233 | 0.05 | 0.93 | OCB | 0.85 | 1 | China |
| Siu Yin Cheunga | 2019 | 282 | 0.04 | 0.93 | Creativity | 0.89 | 1 | China |
| Ben Haobin Ye | 2020 | 531 | 0.57 | 0.9 | Creativity | 0.88 | 0 | China |
| Xin Qin | 2019 | 275 | 0.37 | 0.9 | LMX | 0.83 | 1 | China |
| Xin Qin | 2019 | 275 | -0.21 | 0.9 | Negative affect | 0.85 | 0 | China |
| CHANG-WOOK JEUNG | 2017 | 306 | 0.12 | 0.9 | Voice | 0.86 | 0 | Korea |
| Chang-Wook Jeung | 2016 | 294 | 0.37 | 0.96 | Psychological empowerment | 0.93 | 0 | Korea |
| Xiang Zhou | 2021 | 598 | 0.668 | 0.95 | Affective commitment | 0.93 | 0 | China |
| Xiang Zhou | 2021 | 598 | 0.475 | 0.95 | Voice | 0.93 | 0 | China |
| Jie Li | 2018 | 325 | 0.354 | 0.923 | Organizational identification | 0.872 | 0 | China |
| Jie Li | 2018 | 325 | 0.363 | 0.923 | Voice | 0.845 | 1 | China |
| Jie Zhong | 2019 | 228 | 0.48 | 0.89 | Engagement | 0.93 | 1 | China |
| Jie Zhong | 2019 | 228 | 0.45 | 0.89 | Job satisfaction | 0.75 | 1 | China |
| Xiaoshuang Lin | 2019 | 152 | 0.09 | 0.95 | Voice | 0.9 | 1 | China |
| Yanfei Wang | 2018 | 386 | 0.17 | 0.8 | Creativity | 0.86 | 1 | China |
| Fei Zhou | 2018 | 169 | 0.467 | 0.878 | Creativity | 0.857 | 0 | China |
| Guo Qiuyun | 2020 | 183 | 0.21 | 0.73 | Organizational identification | 0.85 | 0 | China |
| Mudassar Ali | 2020 | 337 | 0.196 | 0.92 | Creativity | 0.91 | 0 | Pakistan |
| Mudassar Ali | 2020 | 337 | 0.201 | 0.92 | psychological empowerment | 0.94 | 0 | Pakistan |
| Juan Wang | 2019 | 382 | 0.39 | 0.9 | Voice | 0.93 | 1 | China |
| Shaohui Lei | 2021 | 348 | 0.501 | 0.936 | Creativity | 0.905 | 0 | China |
| Shaohui Lei | 2021 | 348 | 0.594 | 0.936 | LMX | 0.892 | 0 | China |
| XuHui Li | 2019 | 230 | 0.17 | 0.97 | Voice | 0.92 | 0 | China |
| Kang-Hwa Shaw | 2021 | 553 | 0.48 | 0.92 | Voice | 0.85 | 0 | China |
| Yanhong Chen | 2018 | 286 | 0.37 | 0.94 | psychological empowerment | 0.85 | 0 | China |
| Jun Song | 2022 | 203 | 0.35 | 0.96 | Organizational identification | 0.88 | 0 | China |
| Zhen Liu | 2019 | 345 | 0.507 | 0.872 | Creativity | 0.92 | 0 | China |
| Yue Wang | 2019 | 273 | 0.63 | 0.92 | Affective trust | 0.93 | 0 | China |
| Lianying Zhang | 2022 | 80 | 0.39 | 1 | OCB | 0.69 | 0 | China |
| Lianying Zhang | 2022 | 80 | 0.53 | 1 | Task performance | 0.7 | 0 | China |
| Wasim Abbas | 2020 | 359 | 0.18 | 0.89 | Creativity | 0.84 | 0 | Pakistan |
| Teng-Chu Chiu | 2020 | 433 | -0.02 | 0.92 | OCB | 0.9 | 1 | China |
| Xun Ye | 2019 | 203 | 0.27 | 0.85 | Task performance | 0.77 | 0 | China |
| Bradley P. Owens | 2013 | 704 | 0.25 | 0.97 | Engagement | 0.92 | 0 | US |
| Bradley P. Owens | 2013 | 704 | 0.44 | 0.97 | Job satisfaction | 0.82 | 0 | US |
| Bradley P. Owens | 2013 | 704 | -0.14 | 0.97 | Voluntary turnover | 1 | 1 | US |

Note. We only show the first author and the essential information. In relation to cross-temporal, the cross-temporal is coded as “0”, while the time-lagged is coded as “1”.

**Reference**

Abbas, W., & Wu, W. (2019). The moderating role of intrapreneurial personality in relation between leader humility and innovative behavior. *Human Systems Management, 38*(4), 329-337. doi:10.3233/hsm-190548

Abbas, W., & Wu, W. (2021). Organizational Justice, Leader Humility, and Service Employees’ Innovative Behavior in a Collectivistic Culture: The Case of Pakistan. *Review of Business Management*, 153-179. doi:10.7819/rbgn.v23i1.4094

Al Wali, J., Muthuveloo, R., & Teoh, A. P. (2022). Unravelling the nexus between creative self-efficacy, humble leadership, innovative work behaviour and job performance amongst physicians in public hospitals. *Asia-Pacific Journal of Business Administration*. doi:10.1108/apjba-05-2021-0205

Ali, M., Zhang, L., Shah, S. J., Khan, S., & Shah, A. M. (2020). Impact of humble leadership on project success: the mediating role of psychological empowerment and innovative work behavior. *Leadership & Organization Development Journal, 41*(3), 349-367. doi:10.1108/lodj-05-2019-0230

Asghar, F., Mahmood, S., Iqbal Khan, K., Gohar Qureshi, M., & Fakhri, M. (2021). Eminence of Leader Humility for Follower Creativity During COVID-19: The Role of Self-Efficacy and Proactive Personality. *Front Psychol, 12*, 790517. doi:10.3389/fpsyg.2021.790517

Bahadur, W., & Ali, A. (2021). Linking leader humility with service performance: the role of service climate and customer mistreatment. *Asian Business & Management*. doi:10.1057/s41291-020-00145-9

Basford, T. E., Offermann, L. R., & Behrend, T. S. (2014). Please accept my sincerest apologies: Examining follower reactions to leader apology. *Journal of Business Ethics, 119*(1), 99-117.

Bharanitharan, K., Chen, Z. X., Bahmannia, S., & Lowe, K. B. (2018). Is Leader Humility a Friend or Foe, or Both? An Attachment Theory Lens on Leader Humility and Its Contradictory Outcomes. *Journal of Business Ethics, 160*(3), 729-743. doi:10.1007/s10551-018-3925-z

Bhatia, R., & Bhatia, A. (2020). The psychological effect of humble leadership on employee turnover and resilience. *PalArch's Journal of Archaeology of Egypt/Egyptology, 17*(9), 6827-6856.

Carnevale, J. B., Huang, L., & Paterson, T. (2019). LMX-differentiation strengthens the prosocial consequences of leader humility: An identification and social exchange perspective. *Journal of Business Research, 96*, 287-296. doi:10.1016/j.jbusres.2018.11.048

Chen, L., Zhang, L., Zhang, Z.-D., & Gao, H. (2019). Does emotional resistance to change definitely stifle voice behavior? Revealing the reversal effect of humble leadership. *Current Psychology, 40*(11), 5333-5348. doi:10.1007/s12144-019-00462-z

Chen, Y., Liu, B., Zhang, L., & Qian, S. (2018). Can leader “humility” spark employee “proactivity”? The mediating role of psychological empowerment. *Leadership & Organization Development Journal, 39*(3), 326-339. doi:10.1108/lodj-10-2017-0307

Cheung, S. Y., Huang, E. G., Chang, S., & Wei, L. (2020). Does being mindful make people more creative at work? The role of creative process engagement and perceived leader humility. *Organizational Behavior and Human Decision Processes, 159*, 39-48. doi:10.1016/j.obhdp.2019.12.003

Cho, J., Schilpzand, P., Huang, L., & Paterson, T. (2020). How and When Humble Leadership Facilitates Employee Job Performance: The Roles of Feeling Trusted and Job Autonomy. *Journal of Leadership & Organizational Studies, 28*(2), 169-184. doi:10.1177/1548051820979634

Diao, H., Song, L. J., Wang, Y., & Zhong, J. (2019). Being Passionate to Perform: The Joint Effect of Leader Humility and Follower Humility. *Front Psychol, 10*, 1059. doi:10.3389/fpsyg.2019.01059

Ding, H., Yu, E., Chu, X., Li, Y., & Amin, K. (2020). Humble Leadership Affects Organizational Citizenship Behavior: The Sequential Mediating Effect of Strengths Use and Job Crafting. *Front Psychol, 11*, 65. doi:10.3389/fpsyg.2020.00065

Jeung, C.-W., & Yoon, H. J. (2016). Leader humility and psychological empowerment: investigating contingencies. *Journal of Managerial Psychology, 31*(7), 1122-1136. doi:10.1108/jmp-07-2015-0270

Jeung, C.-W., & Yoon, H. J. (2017). When leadership elicits voice: Evidence for a mediated moderation model. *Journal of Management & Organization, 24*(1), 40-61. doi:10.1017/jmo.2017.42

Lei, S., Peng, L., & Guo, Y. (2021). Investigating the effect of leader humility on subordinates’ service creativity: a moderated dual path model. *Current Psychology*. doi:10.1007/s12144-021-01887-1

Li, J., Liang, Q., Zhang, Z., & Wang, X. (2018). Leader humility and constructive voice behavior in China: a dual process model. *International Journal of Manpower, 39*(6), 840-854. doi:10.1108/ijm-06-2017-0137

Li, J., Liang, Q. Z., & Zhang, Z. Z. (2016). The effect of humble leader behavior, leader expertise, and organizational identification on employee turnover intention. *Journal of Applied Business Research (JABR), 32*(4), 1145-1156.

Li, X., Li, M., Fu, J., & Ullah, A. (2019). Leader humility and employee voice: The role of employees’ regulatory focus and voice-role conception. *Social Behavior and Personality: an international journal, 47*(6), 1-12. doi:10.2224/sbp.7811

Li, X., Xue, J., & Liu, J. (2021). Linking leader humility to employee creative performance: Work engagement as a mediator. *Social Behavior and Personality: an international journal, 49*(6), 1-7. doi:10.2224/sbp.10358

Liborius, P., & Kiewitz, C. (2022). When leader humility meets follower competitiveness: Relationships with follower affective trust, intended and voluntary turnover. *Journal of Vocational Behavior, 135*. doi:10.1016/j.jvb.2022.103719

Lin, X., Chen, Z. X., Tse, H. H. M., Wei, W., & Ma, C. (2017). Why and When Employees Like to Speak up More Under Humble Leaders? The Roles of Personal Sense of Power and Power Distance. *Journal of Business Ethics, 158*(4), 937-950. doi:10.1007/s10551-017-3704-2

LIU, Y.-Y. (2016). A Research on the Impact of Humble Leadership on Employees’ Voice Behavior. *2016 3rd International Conference on Social Science*.

Liu, Z., & Liu, W. (2019). Humble leadership and employee creativity: the mediating role of knowledge hiding. *Management and Economic Journal, 3*(5), 549-557.

Ma, C., Wu, C.-H., Chen, Z. X., Jiang, X., & Wei, W. (2019). Why and when leader humility promotes constructive voice: a crossover of energy perspective. *Personnel Review, 49*(5), 1157-1175. doi:10.1108/pr-02-2019-0049

Mao, J., Chiu, C. Y., Owens, B. P., Brown, J. A., & Liao, J. (2018). Growing Followers: Exploring the Effects of Leader Humility on Follower Self‐Expansion, Self‐Efficacy, and Performance. *Journal of Management Studies, 56*(2), 343-371. doi:10.1111/joms.12395

Nguyen, D. T. N., Teo, S. T. T., Halvorsen, B., & Staples, W. (2020). Leader Humility and Knowledge Sharing Intention: A Serial Mediation Model. *Front Psychol, 11*, 560704. doi:10.3389/fpsyg.2020.560704

Owens, B. P., Johnson, M. D., & Mitchell, T. R. (2013). Expressed Humility in Organizations: Implications for Performance, Teams, and Leadership. *Organization Science, 24*(5), 1517-1538. doi:10.1287/orsc.1120.0795

Qian, S., Liu, Y., & Chen, Y. (2020). Leader humility as a predictor of employees’ feedback-seeking behavior: The intervening role of psychological safety and job insecurity. *Current Psychology, 41*(3), 1348-1360. doi:10.1007/s12144-020-00663-x

Qin, X., Chen, C., Yam, K. C., Huang, M., & Ju, D. (2020). The double-edged sword of leader humility: Investigating when and why leader humility promotes versus inhibits subordinate deviance. *J Appl Psychol, 105*(7), 693-712. doi:10.1037/apl0000456

Qin, X., Liu, X., Brown, J. A., Zheng, X., & Owens, B. P. (2019). Humility Harmonized? Exploring Whether and How Leader and Employee Humility (In)Congruence Influences Employee Citizenship and Deviance Behaviors. *Journal of Business Ethics, 170*(1), 147-165. doi:10.1007/s10551-019-04250-4

Qiuyun, G., Liu, W., Zhou, K., & Mao, J. (2020). Leader humility and employee organizational deviance: the role of sense of power and organizational identification. *Leadership & Organization Development Journal, 41*(3), 463-479. doi:10.1108/lodj-07-2019-0287

Shaw, K.-H., & Mao, J. (2021). Leader–follower congruence in humility and follower voice: the mediating role of affective attachment. *Current Psychology*. doi:10.1007/s12144-021-01475-3

Song, J., Wang, K., & He, C. (2022). The influence of organizational identification on the curvilinear relationship between leader humility and follower unethical pro-organizational behavior. *Journal of Management & Organization*, 1-17. doi:10.1017/jmo.2022.10

Wang, J., Zhang, Z., & Jia, M. (2016). Understanding How Leader Humility Enhances Employee Creativity. *The Journal of Applied Behavioral Science, 53*(1), 5-31. doi:10.1177/0021886316678907

Wang, J., Zhang, Z., & Jia, M. (2019). Echoes of Corporate Social Responsibility: How and When Does CSR Influence Employees’ Promotive and Prohibitive Voices? *Journal of Business Ethics, 167*(2), 253-269. doi:10.1007/s10551-019-04151-6

Wang, P., Dust, S., & Wang, Z. (2021). Leader Sex and Employee Power Distance Orientation as Boundary Conditions of the Relationship between Leader Humility and Leader-Member Exchange. *Human Performance, 34*(5), 351-367. doi:10.1080/08959285.2021.1966632

Wang, X., Liu, Z., Wen, X., & Xiao, Q. (2022). An implicit leadership theory lens on leader humility and employee outcomes: Examining individual and organizational contingencies. *Tourism Management, 89*. doi:10.1016/j.tourman.2021.104448

Wang, X., & Zhou, F. (2021). Managing the Uncertainties Inherent in Prohibitive Voice: How Leadership Interacts With Employee Political Skill. *Front Psychol, 12*, 702964. doi:10.3389/fpsyg.2021.702964

Wang, Y., Liu, J., & Zhu, Y. (2018). How does humble leadership promote follower creativity? The roles of psychological capital and growth need strength. *Leadership & Organization Development Journal, 39*(4), 507-521. doi:10.1108/lodj-03-2017-0069

Wang, Y., Liu, J., & Zhu, Y. (2018). Humble Leadership, Psychological Safety, Knowledge Sharing, and Follower Creativity: A Cross-Level Investigation. *Front Psychol, 9*, 1727. doi:10.3389/fpsyg.2018.01727

Wang, Y., Luo, W., Zhang, J., & Guo, Y. (2019). More humility, less counterproductive work behaviors? The role of interpersonal justice and trust. *Frontiers of Business Research in China, 13*(1). doi:10.1186/s11782-019-0069-7

Yang, J., Zhang, W., & Chen, X. (2019). Why Do Leaders Express Humility and How Does This Matter: A Rational Choice Perspective. *Front Psychol, 10*, 1925. doi:10.3389/fpsyg.2019.01925

Yang, K., Zhou, L., Wang, Z., Lin, C., & Luo, Z. (2019). Humble leadership and innovative behaviour among Chinese nurses: The mediating role of work engagement. *J Nurs Manag, 27*(8), 1801-1808. doi:10.1111/jonm.12879

Ye, B. H., Tung, V. W. S., Li, J. J., & Zhu, H. (2020). Leader humility, team humility and employee creative performance: The moderating roles of task dependence and competitive climate. *Tourism Management, 81*. doi:10.1016/j.tourman.2020.104170

Ye, X. (2019). *Humble leadership and employee performance: Examining a moderated-mediation model.* Paper presented at the 4th International Symposium on Management, Economics, E-business and Marketing.

Zheng, G., Zhou, Y., & Song, L. J. (2022). Linking leader humility with follower performance: A perspective of multi-foci relational identification. *Current Psychology*. doi:10.1007/s12144-022-02966-7

Zhong, J., Zhang, L., Li, P., & Zhang, D. Z. (2019). Can leader humility enhance employee wellbeing? The mediating role of employee humility. *Leadership & Organization Development Journal, 41*(1), 19-36. doi:10.1108/lodj-03-2019-0124

Zhou, F., & Wu, Y. J. (2018). How humble leadership fosters employee innovation behavior. *Leadership & Organization Development Journal, 39*(3), 375-387. doi:10.1108/lodj-07-2017-0181

Zhou, X., Wu, Z., Liang, D., Jia, R., Wang, M., Chen, C., & Lu, G. (2021). Nurses' voice behaviour: The influence of humble leadership, affective commitment and job embeddedness in China. *J Nurs Manag, 29*(6), 1603-1612. doi:10.1111/jonm.13306
